# Contribution of endophytes towards improving plant bioactive metabolites: a rescue option against red-taping of medicinal plants

**DOI:** 10.3389/fpls.2023.1248319

**Published:** 2023-09-12

**Authors:** Sinawo Tsipinana, Samah Husseiny, Kazeem A. Alayande, Mai Raslan, Stephen Amoo, Rasheed Adeleke

**Affiliations:** ^1^ Unit for Environmental Sciences and Management, North-West University, Potchefstroom, South Africa; ^2^ Department of Biotechnology and Life Sciences, Faculty of Postgraduate Studies for Advanced Sciences, Beni-Suef University, Beni-Suef, Egypt; ^3^ Agricultural Research Council – Vegetables, Industrial and Medicinal Plants, Roodeplaat, Pretoria, South Africa

**Keywords:** over-harvesting, secondary metabolites, endangered species, heavy metal contaminant, co-cultivation

## Abstract

Medicinal plants remain a valuable source for natural drug bioprospecting owing to their multi-target spectrum. However, their use as raw materials for novel drug synthesis has been greatly limited by unsustainable harvesting leading to decimation of their wild populations coupled with inherent low concentrations of constituent secondary metabolites per unit mass. Thus, adding value to the medicinal plants research dynamics calls for adequate attention. In light of this, medicinal plants harbour endophytes which are believed to be contributing towards the host plant survival and bioactive metabolites through series of physiological interference. Stimulating secondary metabolite production in medicinal plants by using endophytes as plant growth regulators has been demonstrated to be one of the most effective methods for increasing metabolite syntheses. Use of endophytes as plant growth promotors could help to ensure continuous supply of medicinal plants, and mitigate issues with fear of extinction. Endophytes minimize heavy metal toxicity in medicinal plants. It has been hypothesized that when medicinal plants are exposed to harsh conditions, associated endophytes are the primary signalling channels that induce defensive reactions. Endophytes go through different biochemical processes which lead to activation of defence mechanisms in the host plants. Thus, through signal transduction pathways, endophytic microorganisms influence genes involved in the generation of secondary metabolites by plant cells. Additionally, elucidating the role of gene clusters in production of secondary metabolites could expose factors associated with low secondary metabolites by medicinal plants. Promising endophyte strains can be manipulated for enhanced production of metabolites, hence, better probability of novel bioactive metabolites through strain improvement, mutagenesis, co-cultivation, and media adjustment.

## 1 Introduction

Several clinical conditions, ranging from acute to chronic, have long plagued mankind since the dawn of time (Takahashi et al., 2020). Traditionally, medicinal plants have been used as remedies to sustain human health and well-being (Palanichamy et al., 2018). Many of such plants are essential sources and reservoirs of bioactive metabolites used in production of pharmaceuticals for therapeutic purposes. However, several of these medicinal plants have been red-taped and classified as threatened species by different conservative authorities (Chen et al., 2016) due to unabated demand, aggressive wild harvesting, habitat degradation, and ultimate fear of extinction.

Mass production of bioactive compounds from medicinal plants often requires a high volume of biomass. The quality and quantity of naturally synthesized bioactive compounds fluctuate with varying environmental growth conditions, compounded by the global climate change (Venugopalan and Srivastava, 2015; Singh et al., 2018). Thus, there is a growing need for sustainable alternative templates or sources for efficient production of drugs to meet the ever-increasing demands (Asmita and Sashirekha, 2016). Specifically, different strategies have been explored on improving quality and quantity of bioactive secondary metabolites associated with medicinal plants (Bourgaud et al., 2001).

Moreover, the global challenge of antibiotic resistance coupled with recurrent, new and re-emerging life-threatening infectious diseases necessitates dedicated search for novel therapeutic and prophylactic agents (Golinska et al., 2015). A deliberate attention has therefore been focused on the metabolites produced by varieties of bacterial and fungal endophytes (Ek-Ramos et al., 2019). The endophytes co-exist with medicinal plants and as well exhibit pharmacological activities similar to those of the host plants (Palanichamy et al., 2018). This positions endophytes as potential resources for bioactive natural products which could eventually play a significant role in the discovery of new drugs (Manandhar et al., 2019). They are known for their unique ability to influence the quality of bioactive secondary metabolites produced by their hosts, hence, a better understanding of the interactions between medicinal plants and their symbiont endophytes is paramount (Jia et al., 2016).

Furthermore, the complex nature of secondary metabolite production pathways could be a limiting factor for diversity of natural products (Thomford et al., 2018). Advanced technologies can enable the continuous manufacturing of high-valued bioactive compounds with short production cycles (Marchev et al., 2020). Sensitivity of new analytical instrument for compound identification, elucidation and quantification has greatly increased. This allows for study of important bioactive compounds even at very low concentrations, and boosting the likelihood of discovering new compounds that could have escaped detection due to limitations from the previous analytical techniques. Slow-growing trees and shrubs produce most of the high-valued and often demanded plant-derived pharmaceuticals, but at very low rate both in the *in vivo* field environments and *in vitro* tissue culture set-ups. Therefore, application of modern biotechnology would definitively play a critical role in the production of therapeutic plant-based metabolites at industrial and commercial scale, while ensuring protection of nature reserves (Isah et al., 2018). Synthesis of diverse varieties of phytochemicals have been observed in undifferentiated plant tissue cultures under controlled laboratory condition while induced with elicitors or feeding precursors. However, this has not been without limitations. For instance, introduction of the genes that are involved in biosynthesis of artemisinin in the heterologous hosts ended up in a very low yield (Guerriero et al., 2018).

Secondary metabolite biosynthesis involves a conglomerate of genes, enzymes, and metabolic pathways, most of which are not well understood due to their complexity (Castillo et al., 2013). For instance, genes are sometimes not expressed in a functional state which makes it impossible to determine how much of a role they play in biosynthesis of secondary metabolites (Kroymann, 2011). In addition, gene-metabolites networks remain unclear because most functional genes in medicinal plants remain unknown and this constitutes a major constraint in the upscaling of metabolite production (Yang et al., 2014). Consequently, many metabolic pathways are not discovered due to the unknown functional genomic (Yang et al., 2014). Currently there are only a few assembled genomes of medicinal plants that address the function of genes involved in the synthesis of secondary metabolites (Chakraborty, 2018).

In addition, plant cytochrome P450s enzymes, for example, are one of the keystone enzymes for the biosynthesis of secondary metabolites such as terpenoids, glucosinolates, phenylpropanoids, and alkaloids, yet a number of their catalytic functions remains unknown (Giddings et al., 2011). Plants contain a large number of P450 genes, and identifying a P450 that catalyzes a specific metabolic transformation in plants remains difficult due to P450 gene similarities and unknown catalytic activities (Nguyen and Dang, 2021.). *Catharanthus roseus*, for example, is one of the sources of anti-cancer drugs, and produces compounds such as the alkaloid tabersonine. However, only 5 *Catharanthus roseus* plant P450s (cinnamate 4-hydroxylase, flavonoid 3′,5′-hydroxylase, secologanin synthase, geraniol 10-hydroxylase, and tabersonine 16-hydroxylase) have been successfully characterized for the biosynthesis of plant secondary metabolites such as alkaloids (Giddings et al., 2011; Chakraborty, 2018).

This review, therefore, highlights importance of medicinal plants in folklore remedy and how modern techniques can be employed to improve their biomass and quality of bioactive secondary metabolites they produce through exploring and enhancing contributions of the symbiont endophytes in support of the host plants. This is with a view to reducing threat of extinction due to over-harvesting of the medicinal plants, thereby supporting genuine conservation efforts for sustainable planet.

## 2 Medicinal plants in traditional healing systems

Medicinal plants contain several compounds of therapeutic and prophylactic importance; hence, man have relied on them traditionally in the management and prevention of different ailments ranging from mild to more serious medical conditions (Hussein and El-Anssary, 2019). Most of these plants are limited in distribution and characterized with localized indigenous knowledge associated with their applications. In terms of abundance, China and India are the top two nations with the most medicinal plants, followed by Colombia, United States and then South Africa (Chen et al., 2016). A growing number of people in developing countries rely on medicinal plants for their primary healthcare (Jain et al., 2019). The historic records on the use of medicinal plants for the treatment of ailments dated back to ~5000 years ago on a Sumerian clay slab from Nagpur (Bernardini et al., 2018; Suntar, 2020). It contained information on over 100 plant-derived pharmaceutics and oils obtained from various herbal plants (Wanjari and Wanjari, 2019; Suntar, 2020). These medicinal plants are mostly used in their raw state, either by infusion or decoction (Suntar, 2020). Studies on the extraction and identification of chemical components from these therapeutic plants dated back to 1800s, and morphine was the first biochemical compound to be extracted from plants (Bernardini et al., 2018; Suntar, 2020). In addition, extracts from medicinal plants have proven to be the backbone for modern drug syntheses while their bioactive secondary metabolites are primarily responsible for this effectiveness (Hussein and El-Anssary, 2019; Jain et al., 2019).

## 3 Production of secondary metabolites and their medicinal importance

All plants undergo primary metabolism, while the metabolites produced are crucial for their growth and development (Sinha et al., 2019), and serve as precursors for the synthesis of secondary metabolites (Nielsen and Nielsen, 2017). For instance, carbon assimilated during photosynthesis serves as a substrate for secondary metabolism. The slow plant development under stress conditions often increases production of secondary metabolites as more carbon would be available for this purpose (Van Wyk and Prinsloo, 2020). It has also been noted that plants containing large contents of nitrogen tends to be prime producers of nitrogenous secondary metabolites such as alkaloids (Puri et al., 2018).

Most secondary metabolites in plants are produced as defence mechanism in response to unfavourable conditions (Cardoso et al., 2019). Secondary metabolites can be divided into a few classes based on their functional groups, and these include alkaloids, phenolics, flavonoids, saponins, tannins, lignins, steroids, terpenoids, anthocyanins, tetralones, cardiac glycosides, and anthraquinones (Guerriero et al., 2018). Plants and microorganisms utilise three pathways for secondary metabolite biosynthesis; shikimate, polyketide, and mevalonate (Kumara et al., 2014; Burragoni and Jeon, 2021). Shikimate is primarily responsible for the synthesis of aromatic amino acid compounds, whereas polyketide and mevalonate pathways are mainly responsible for generation of molecules having medicinal properties (Kumara et al., 2014).

The largest group of secondary metabolites is alkaloids, and they are found in almost 20% of vascular plants (Mahajan et al., 2020). Alkaloids are hydrophilic and are usually stored in the vacuoles (Yang L. et al., 2018). They are mainly produced through decarboxylation of amino acids such as tryptophan, tyrosine, histidine, lysine and ornithine using different pathways (Hussein and El-Anssary, 2019; Burragoni and Jeon, 2021). They have a number of therapeutic applications including anticancer and antimalarial activities (Burragoni and Jeon, 2021; Keshri et al., 2021). It has been discovered that there are more than 20,000 alkaloids that can be extracted from plants. They are also present in a variety of microbes, with *Bacillus* species being notable producers of alkaloids (Burragoni and Jeon, 2021).

The second largest group is phenol and are produced via shikimate pathway. They are best known as dietary metabolites with a wide range of therapeutic applications such as anti-inflammatory, anti-allergic, anti-cancer, anti-viral, antioxidant and anti-ulcer properties (Jamwal et al., 2018; Burragoni and Jeon, 2021). Phenolic compounds also play a vital role in plant pigmentation (Yang S. Q. et al., 2018). Terpenes, which are another prevalent group, are categorized into three main subgroups, monoterpenes, sesquiterpenes and di-terpenes based on the number of carbon isoprene units they contain (Awuchi, 2019). Terpenes are normally biosynthesised from acetyl-CoA and/or glycolytic intermediates and are lipophilic. There are two enzymatic pathways, mevalonic acid and methylerythritol 4-phosphate pathways that are involved in the production of terpenes (Hussain et al., 2012). Artemisinin is a well-known sesquiterpene metabolite produced by *Artemisia* species and it has been found to be effective in controlling cancer, malaria, and several infectious diseases (Pandey and Pandey-Rai, 2016; Guerriero et al., 2018). Taxol is also a diterpenoid anticancer medication that is commonly used to suppress the growth of various malignancies (Yan et al., 2018), and it was first extracted from *Taxus brevifolia* (Guerriero et al., 2018). Thereafter, it was recommended that terpene compounds be included in daily human dietary as a prophylactic measure against a variety of diseases (Palanichamy et al., 2018). All these secondary metabolites have contributed significantly to the development of medications from medicinal plants.

## 4 Uniqueness and valuable attributes of plant-based natural drugs

Over the years, there has been rising global demand for natural products from medicinal plants and herbs which has contributed to their enhanced market value (Williams et al., 2013). The increased demand for medicinal plants is linked to increasing rate of communicable and non-communicable chronic diseases such as diabetes, cardiovascular, neurodegenerative, and cancer diseases (Espín et al., 2007; Takahashi et al., 2020). Pathogenic microorganisms are getting more resistant to antibiotics leading to multi-drug resistant strains becoming a global public health crisis (Mohammadi et al., 2020). Antibiotic resistance is estimated to cause more than 700,000 fatalities worldwide annually, with a prediction that the trajectory could be doubled by 2050 if proper measures are not implemented (Ye et al., 2020).

That being the case, there is a rising demand for novel therapeutic agents to tackle infections, and natural products have gained popularity in combating multi-drug resistance challenges (Ye et al., 2020). Despite a global drop in malaria-related deaths in recent years, sub-Saharan African countries continue to have the highest rate of malaria-related fatalities (Okell et al., 2023). *Plasmodium falciparum* has developed resistance to several anti-malarial medications, and this is prevalent mainly in Southeast Asian and sub-Saharan countries (Dadgostar, 2019). However, natural products such as artemisinin, have exhibited significant efficacy against multidrug-resistant strains of malaria parasites with minimal side effects (Gupta et al., 2020; Mohammadi et al., 2020). Drugs of natural origin have demonstrated high level of potency and cost effectiveness (Banyal et al., 2021). They constitute almost half of the available medications (Banyal et al., 2021).

Despite the strong demand for natural-based drugs, the rate of discovery of novel metabolites is quite low (Gupta et al., 2020). There are more than half a million medicinal plants globally in which most of them have not been exploited for their therapeutic potentials (Dar et al., 2017; Anand et al., 2019). Most of the commercialised high-valued secondary metabolites with minimal or no toxicity are of plant origin (Nomura et al., 2018). Attempts to identify and synthesize drugs from medicinal plants have reduced drastically (Scotti et al., 2017). Some of the main reasons for this include (i) medicinal plant population decimation leading to the extinction of some species, (ii) generally low quantity of secondary metabolites produced by medicinal plants, and (iii) access to modern and sophisticated analytical equipment for novel discovery from the crude components. Undoubtedly, there are great potentials for more drug discoveries because only ~15% of the existing medicinal plants have been explored for their potential as a source of therapeutic agents (Mathur and Hoskins, 2017).

Hence, adding value to the medicinal plant research dynamic would be another way to ensure the continuous use of medicinal plants in the search for new medications. There have been a paucity of investigations into the elements that influence medicinal plant secondary metabolite production, as well as prospective tactics for increasing their production. For example, medicinal plants harbor a variety of endophytes capable of producing secondary metabolites similar to those of their host plants. Thus, new paradigms including discovery of endophyte roles in the survival and defence tactics of their host plants are essential.

## 5 Endophytes intervention towards improving limitations associated with medicinal plant use

**Figure 1:**
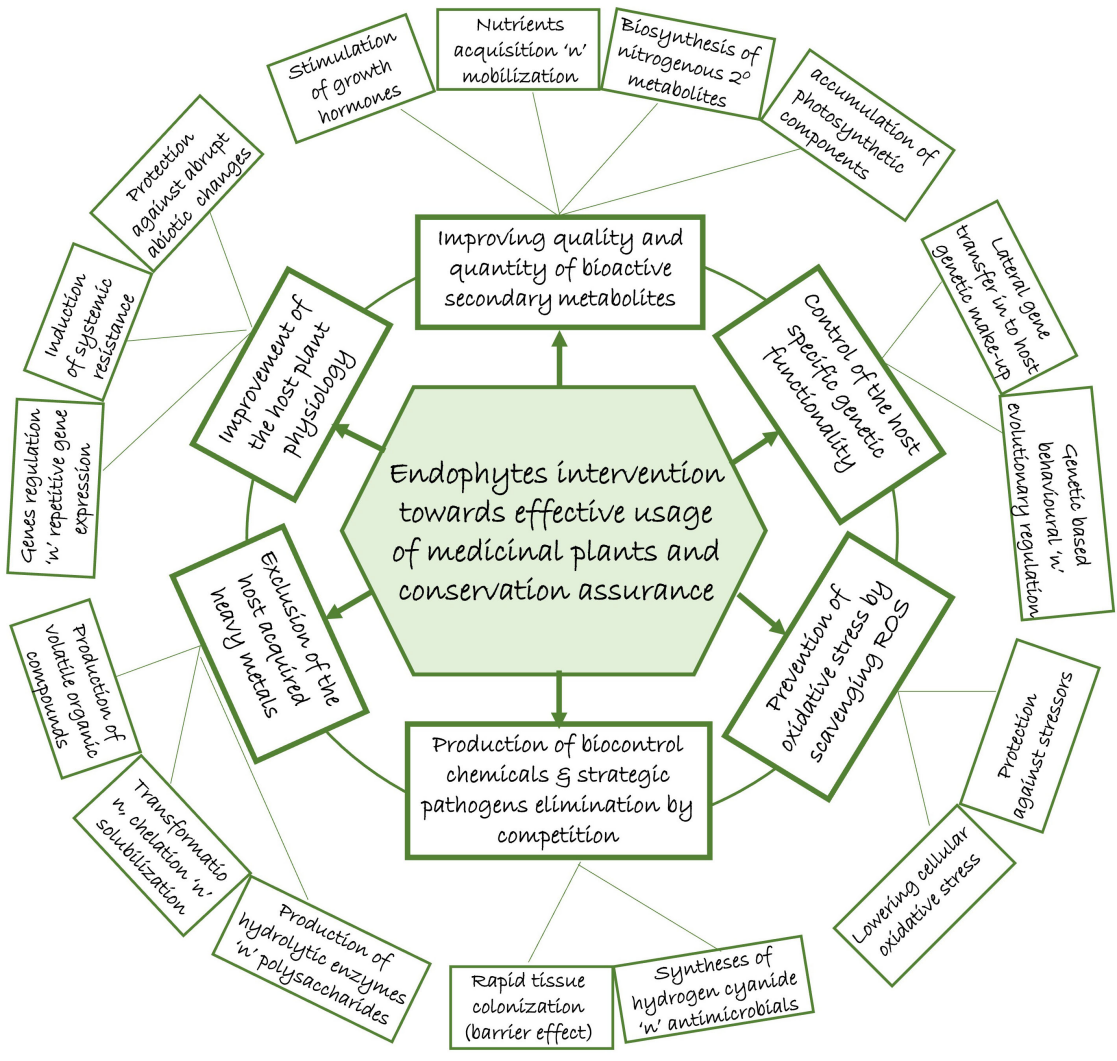
Potentials of endophytes in mitigating the over-reliance impact on medicinal plants.

Endophytes as symbiont microbial community in the tissue of plants, offer a number of inherent potential benefits towards sustaining the mutual association (**Figure 1**). If consciously explored, the contributions by endophytes could become manipulative templates which could help mitigate the impact of over-harvesting due to over-reliance on medicinal plants, most importantly in folklore remedy. This could reduce the fear of extinction that has prompted strict regulations (red tapping) by the conservationists.

### 5.1 Endophytes ameliorating factors responsible for medicinal plants extinction

Flowering medicinal plants range from 50,000 – 80,000 among which about 15,000 of them are threatened by extinction as a result of over harvesting, deforestation and recurrent wildfires (Chen et al., 2016; Maury et al., 2020). Transition from subsistence to commercial trading of medicinal plants has been identified as one of the primary causes of their overharvesting from the wild (Williams et al., 2013). It is also encouraged by the high demand due to growing human population, as huge quantities are required to meet the market demand. It has been projected that loss of several species of medicinal plants is at its peak with possible loss of a number of potential drugs. This threat has been known for decades, but not much work has been done to save the plant biodiversity (Chen et al., 2016; Shafi et al., 2021).

Climate change is another factor affecting the distribution and abundance of medicinal plants with high capability to cause extinction (Gairola et al., 2010). According to reports from the Intergovernmental Panel on Climate Change (IPCC), global average temperatures have risen and this will have a significant impact on plant diversity (Anderson et al., 2020). Each degree Celsius rise in average temperature can have a negative impact on medicinal plant distribution, as these changes are likely to increase plant mortality and extinction risk (Tangjitman et al., 2015). The impact of climate change on certain plants becomes more severe as some plants take longer to adapt to changes in environmental conditions (Akula and Ravishankar, 2011). Plant conservation is critical because it seeks to preserve plant species, especially those that are endangered, in order to ensure a steady supply of essential products while also improving plant biodiversity (Das et al., 2016; Maury et al., 2020).

A variety of measures have been put in place with the intention of conserving nature and guaranteeing a stable supply of vital and vulnerable medicinal plants for drug production (Guerriero et al., 2018). Using *in-situ* and *ex-situ* conservation strategies are among the most effective ways to preserve medicinal plants (Chen et al., 2016; Kadam and Pawar, 2020). Some medicinal plants are site- and environment-specific in their ability to produce significant secondary metabolites, and they might not be able to do so under controlled environments (Wani et al., 2021). In such cases, it is advised to practice *in-situ* conservation of such medicinal plants, which means preserving medicinal plants in their natural habitats (Rajpurohit and Jhang, 2015). *Ex-situ* conservation refers to the preservation of medicinal plants away from their natural habitats (Wani et al., 2021). Unfortunately, several of these techniques have shown to be insufficient for improving the quality and quantity of secondary metabolites from medicinal plants, although adequate for plant conservation.

Finding techniques to improve medicinal plants bioactive chemical production is of utmost importance (Isah et al., 2018). Awasthi et al. (2011) noted that a combination of compatible arbuscular mycorrhizal fungi, *Glomus mosseae* and *Bacillus subtilis* endophytic microbes increased the production of artemisinin content in the leaves of *Artemisia annua* L. In addition, endophytes are essential growth promoting agent in plants (Golinska et al., 2015). They are a group of microorganisms, mainly bacteria and fungi, that colonize the inter- and intracellular tissues of plants without causing apparent diseases (Gouda et al., 2016). Endophytic microbes directly enhance plant growth through acquisition and mobilization of essential nutrients by fixing biological nitrogen, solubilizing insoluble phosphate, and producing siderophrores (Santoyo et al., 2016 Cueva-Yesquén et al., 2021). They further stimulate production of hormones such as indole acetic acid (IAA), gibberellin, cytokinins, ethylene and abscisic acid (Yan et al., 2018; Ghasemnezhad et al., 2021). In a study conducted by Khalil et al. (2021), fungal endophytes (*Penicillium crustosum* EP-2, *Penicillium chrysogenum* EP-3, and *Aspergillus flavus* EP-14) isolated from *Ephedra pachyclada* leaves demonstrated plant growth promoting characteristics such as IAA production. Endophytes indirectly enhance plant growth by protecting plants from biotic and abiotic stressors. For instance, siderophores produced can enhance plant adaptability and tolerance to stress conditions caused by diseases, insects, pests/nematodes while assisting with nutrient acquisition (Dhuldhaj and Pandya, 2021; Sun et al., 2022).

Endophytes have been proven to increase the biosynthesis of nitrogen-containing secondary metabolites such as alkaloids by altering the biochemistry of medicinal plants through nutrient intake and exchanges (Chadha et al., 2015). It has been postulated that nitrogen-fixing endophytes contribute greatly into increasing production of such secondary metabolites by medicinal plants (Tor et al., 2009). Zhang et al. (2013) investigated the ability of *Mycena* sp. to enhance *Anoectochilus formosanus* production of flavonoid and kinsenoside in a greenhouse environment. Song et al. (2017) also noted that using *Bacillus altitudinis* KX230132.1 as an elicitor greatly boost the content of ginsenoside, a bioactive component found in ginseng.

Improved plant biomass could be associated with improved production of secondary metabolites. This is premised on the fact that about 90% of plant dry matter are derived from photosynthetic carbon fixation, and organic matters build-up by photosynthesis which serves as the precursors for secondary metabolite production (Li et al., 2023). Endophytes aid in the accumulation of photosynthetic components of the plant by enhancing the growth of medicinal plants, resulting in a high biosynthesis of secondary metabolites by plants (Gupta and Chaturvedi, 2019; Li et al., 2023). Moreover, several studies have reported degrees of influence the environmental and biological stressors as well as developmental factors have on the timing of flowering in plants (Jagadish et al., 2016; Kazan and Lyons, 2016). Plants often accelerate the flowering process as an indication of stress escape strategy (Bernal et al., 2011), and medicinal plants are not an exception. Synthesis of bioactive secondary metabolites which is the hallmark of the medicinal plants are often triggered in reaction to these stress factors. Interactions between endophytes and the host medicinal plants are equally believed to trigger flowering processes (Khan A. L. et al., 2017). The flowering stage of some medicinal plants is critical to the developmental state with notable impact on the synthesis of secondary metabolites, and production of secondary metabolites is expected to be at its peak (Li Y. et al., 2020). Thus, during this time, harvesting medicinal plants for therapeutic purposes is recommended, depending on the metabolite accumulation site and the plant part(s) used for medicinal purposes.

### 5.2 Endophytes as a shield for medicinal plants against undesirable environmental impacts

Production of secondary metabolites by plants is generally low, about less than 1% of plant dry weight depending on plant’s physiological and developmental stage (Guerriero et al., 2018). Unfavourable environmental conditions result in reduced chances for plant survival (Kliebenstein and Osbourn, 2012). Consequently, environmental changes and/or climate change induced stress are the main determinants for medicinal plant secondary metabolite biosynthesis fluctuations (Verma and Shukla, 2015). In addition, medicinal plant prevalence differs under different climatic conditions and their ability to produce certain metabolites is largely affected by climate variables such as temperature and rainfall (Das et al., 2016; Li et al., 2023). Understanding the biological controls of secondary metabolism in relation to changing climate is essential (Akula and Ravishankar, 2011).

Though medicinal plants may evolve defense mechanisms to cope with high or low temperatures, such mechanisms are often insufficient, resulting in unstable production of secondary metabolites (Chadha et al., 2015; Li T. et al., 2020). For instance, during extremely low temperature, plants channel their metabolism energy through the development of cryo-protective compounds such as sugar alcohols, soluble sugars, and low-molecular nitrogenous compounds at the expense of essential secondary metabolites (Thakur et al., 2019). On the other hand, elevated temperatures are known to promote the production of some secondary metabolites while reducing the production of others, mainly volatile ones, due to volatilization (Gairola et al., 2010). Furthermore, temperature influences some of the primary metabolic processes of plants, such as photosynthesis, respiration, transpiration, and dry matter partitioning, all of which are prerequisites for secondary metabolism (Nahar et al., 2015). The adaptation process that medicinal plants go through under various conditions also causes changes in plant morphology, anatomy and physiological processes (Li T. et al., 2020). All these have an impact on the accumulation of secondary metabolites, therefore, depending on the pathways and response genes that were engaged under various conditions, different metabolites can be biosynthesized by medicinal plants (Li T. et al., 2020).

Medicinal plant associated endophytes on the other hand, are shielded from abrupt changes in the environment (Papik et al., 2020; Wu et al., 2021). Consequently, it has been hypothesized that when medicinal plants are exposed to harsh conditions, the harbouring endophytes are the primary signalling channels that induce a defensive reaction (Singh J. et al., 2019). Endophytes go through different biochemical process which leads to the activation of their defence mechanisms including those of their host plants (Ogbe et al., 2020). These processes include the synthesis of antioxidants, phytohormones, and activation of stress-induced genes, all of which play a part in secondary metabolite synthesis (Ogbe et al., 2020). In order to trigger the response in plants, endophytic signal transduction pathway first binds extracellular signaling molecules and ligands on the cell surface or inside the cell (Jin et al., 2016). Thus, using activating signal transduction pathways, endophytic microorganisms influence genes involved in the generation of secondary metabolites by the plant cells (Li et al., 2023). Moreover, they help medicinal plants flourish by lowering cellular oxidative stress brought on by biotic and abiotic stress factors (Khan A. N. et al., 2017). The ability of medicinal plants to withstand biotic and abiotic stresses might not be from the plants themselves but rather from the associated endophytic microbes (Chadha et al., 2015).

Extreme climatic conditions can also result in disease, insect, and pathogen infestations, and these biotic stressors have substantial impact on the quality and quantity of secondary metabolites produced by medicinal plants. Pathogenic microbes in medicinal plants induce infection and offer a significant risk to patients (Chadha et al., 2015; Slama et al., 2021). However, controlling some plant pathogenic diseases such as vascular wilt is challenging (Yadeta and Thomma, 2013). On the other hand, the continued use of chemical pesticides to combat plant pathogens has led to the emergence of pesticide-resistant pathogenic strains (Hawkins et al., 2019). Medicinal plants activate reactive oxygen species (ROS) and reactive nitrogen species (RNS), which are the primary plant defense responses to combat entry of both beneficial and non-beneficial microorganisms (Kapoor et al., 2019). However, endophytes are capable of degrading both the ROS and RNS through enzymatic actions in order to pave way for the infestation and colonization of their host plants (Chadha et al., 2015). Plant cell walls, including those of medicinal plants, get re-reinforced shortly after endophytic colonization, and this aids in preventing pathogenic infestation. Endophytes also shield medicinal plants from pathogenic attack through a phenomenon known as the “barrier effect,” which occurs due to their widespread and intense replication in the colonized plants, leaving little to no space for non-beneficial microorganisms (Mengistu, 2020).

The use of endophytes as a biological control measure has been recognized to be most effective in controlling plant diseases and pests as endophytes have the ability to antagonize pathogens (Mohamad et al., 2018). An *in-vitro* experiment conducted by Mohamad et al. (2020) revealed that *Bacillus* and *Enterobacter* species isolated from *Thymus vulgaris* were able to significantly promote growth of tomato (*Solanum lycopersicum* L) under saline conditions (NaCl supplemented). The strains were also able to antagonise *Fusarium oxysporum.* The ability of the tomato to withstand stressful conditions was attributed to the plant’s enzymatic activity which was believed to have been initiated by those endophytic strains. In addition, the ability of those endophytes to assist tomato with tolerance to stress was due to their ability to produce a variety of compounds such as nonadecane, tetracosane, cyclohexanecarboxylic acid, 2-, which are antimicrobials.

Endophytes can also control pathogens through a variety of mechanisms, including (i) the synthesis of bio-control chemicals such as siderophore, hydrogen cyanide, and antibacterial components, (ii) competing for important resources, and (iii) induction of systemic resistance via activation of stress signaling hormones like jasmonate and ethylene (Kushwaha et al., 2020; Slama et al., 2021). This was evident in an experiment conducted by Karthikeyan et al. (2012) under saline conditions, where *Achromobacter xylosoxidans* endophyte was able to enhance growth, reduced level of ethylene, and increased metabolic production of antioxidant enzymes by *Catharanthus roseus*. Yang et al. (2015) also reported induced systemic resistance by *Bacillus amyloliquefaciens* isolated from *Ginkgo biloba* which inhibits pepper phytophthora blight. In another study, hydrogen cyanide producing bacterial endophytes isolated from *Panax ginseng* belonging to *Xanthomonadaceae, Paenibacillaceae, Pseudomonadaceae, Micrococcaceae*, and *Bacillacea*, successfully controlled plant fungal pathogens, *Botrytis cinerea* and *Cylindrocarpon destructans* (Hong et al., 2018).

Restricting access to iron by microbial pathogens appears to be a promising strategy for combating infectious diseases in plants (Mohamad et al., 2018). This is often accomplished through microbial production of siderophores. Siderophores are small iron (Fe^3+^) chelating ligands with less than 10 kDa molecular weight and are produced by different microorganisms under iron-deficient conditions (Roskova et al., 2022). The endophytes with high affinity for iron are excellent siderophore producers because they scavenge iron from the surroundings, thus making it unavailable for the pathogens. Hydroxamate is one of the most abundant siderophore secreted by various species of fungi and bacteria (Dhuldhaj and Pandya, 2021). Certain extracellular enzymes produced by the endophytes such as dehydrogenase, β-glucanase, amylases, gelatinase, cellulases, lipases, pectinase, proteinase, and chitinases also protect plants from pathogenic attack through microbial cell wall degradation (Kushwaha et al., 2020; Slama et al., 2021). They further help in strengthening plants and endophytes cell wall boundaries which ultimately build resistance against the pathogenic microbes (Fadiji and Babalola, 2020).

### 5.3 Endophytes reducing uptake of heavy metal contaminants by the plants

Heavy metal toxicity is one of the major problems affecting the use of medicinal plants as raw materials. Heavy metals are usually transmitted from the soil to the plants, affecting the overall health of the plants and their ability to produce important secondary metabolites (Street, 2012). Their presence in non-tolerant plants often lead to a decrease in enzyme activity of the plants which is the backbone for production of secondary metabolites (Sharma and Kumar, 2021). Heavy metal toxicity causes plants to accumulate more ROS (Zheng et al., 2023). Even though ROS are produced as normal by-products of metabolism, different conditions can result to excessive production of ROS which exceeds the defense mechanisms, leading to plant oxidative stress and ultimate cell death (Sharma et al., 2012). Although ROS can result in increased secondary metabolite production, excessive ROS can have a deleterious impact on secondary metabolite production through damage to primary metabolites such as lipids, proteins and nucleic acid compounds, which are the building blocks for secondary metabolites (Sharma et al., 2012). Endophytes on the other hand, are known to aid in the elimination of ROS in plants by scavenging active oxygen systems, which are typically triggered when plants are stressed (Zheng et al., 2023). Endophytes limit the uptake of harmful heavy metals by plants and/or immobilize them through a variety of mechanisms, including transformation, chelation, solubilization, and precipitation (Singh et al., 2020; Durand et al., 2021). For instance, they use a mechanism called biosorption, which includes a number of procedures like ion exchange, electrostatic contact, precipitation, and redox reaction, to capture these dangerous heavy metals and absorb them into their cell walls (Kushwaha and Kashyap, 2021).

Endophytes also protect medicinal plants from heavy metals by producing volatile organic compounds (VOCs) such as terpenoids and phenylpropanoids (Slama et al., 2021). Moreover, endophytes produce a wide range of valuable hydrolytic enzymes which can sequester various organic and inorganic compounds (Suryanarayanan et al., 2012; Liu et al., 2021). Several microbes have been reported for degrading and immobilizing hazardous heavy metals to less-toxic forms (Kushwaha and Kashyap, 2021; Priyadarshini et al., 2021). Microbial enzymes have therefore been demonstrated to have outstanding unique features that many chemical catalysts might not have. Endophytes help medicinal plants with improvement of physiological responses and immune activity (Khan A. N. et al., 2017). Endophytic 1-aminocyclopropane-1-carboxylic acid deaminase (ACC), for example, aids plant tolerance under stress condition by counteracting ethylene inhibitory effects while enhancing plant growth and development (Kushwaha et al., 2020; Zheng et al., 2023).

Lastly, even though heavy metals can enter through other parts of the plant, the root is regarded as their main entry point. Fungi have been proven to be the most prominent in heavy metal and other toxic compounds remediation. The hyphae from fungal microbes help in amelioration of heavy metals through cell wall thickening and increased surface area, which assist in improved absorption of those toxic elements to their cell wall (Nandy et al., 2020; Sharma and Kumar, 2021). Endophytic cell walls also synthesize polysaccharides such as hydroxyl, carboxyl, and amino groups, which function as a barrier to metal ions by increasing the binding sites with positive metal compounds (Zheng et al., 2023).

### 5.4 Endophytes controlling gene clusters in the host plants for biosynthesis of novel active agents

Finding new novel bioactive compounds to treat diverse ailments is a fundamental goal of all drug discovery initiatives. To combat newly emerging and old incurable diseases, new bioactive compounds must be discovered. Despite the strong demand for natural drugs from medicinal plants, the rate of discovery of novel metabolites for treatment of diseases and infections is on decline (Gupta and Chaturvedi, 2019). In the past few years, there has been high probabilities of extracting already known therapeutic components with little to no luck in discovering new compounds for potential new drug discovery from medicinal plants (Li and Vederas, 2009; Dias et al., 2012). The extraction of new and sophisticated metabolites is limited when using the traditional extraction methods only, hence the problems with repeated rediscovery of the same secondary metabolites predominates (Roopashree and Naik, 2019). Medicinal plants contain complex compounds often in relatively small quantities that may be difficult to extract/isolate and identify, hence their capability may be under-investigated (Thomford et al., 2018).

Production of novel natural drugs requires development of innovative multidisciplinary drug discovery methods beyond traditional techniques (Li and Lou, 2018; Zhang and Elliot, 2019). For instance, the employment of high-throughput and molecular models have proven to be effective in the discovery and identification of bioactive secondary metabolites from both plants and microbes (Tawfike et al., 2019; Prasanna, 2022). Historically, identification of secondary metabolites from plants and microorganisms have been reliant on approaches such as traditional culture-based methods, biochemical screening employing GC/LC-MS, HPLC, and NMR (Tiwari and Bae, 2022). However, it has been demonstrated that integrating low- and high-throughput genomic approaches has the ability to preserve the therapeutic novelty of medicinal plants and they can aid in supplying a direction for the optimization of secondary metabolite production for higher yields (Suntar, 2020). However, there is a shortage of studies on the potential use of genome mining approaches to estimate the pathways for biosynthesis of novel secondary metabolites by medicinal plants (Rivera-Chávez et al., 2022). The interaction between endophytes and plants is complex and sometimes misunderstood, but evaluating, identifying, and characterizing genes involved in their mutual association can be helpful in unravelling the underlying mechanisms between the two (Isah, 2019).

Plant-endophyte symbiosis is driven by cohabitation and co-evolution which ultimately leads to a stronger mutualism that becomes engraved with influence on the expression of certain traits in the genetic makeup of both partners (Mengistu, 2020). These endophytes can obtain and transfer certain genes horizontally with the host plants under specific metabolic pathways during colonisation (Gohain et al., 2020). After endophytes colonize their host plant, a number of genes regulate their behavior, resulting in changes in their genotypic features as well as that of the plants (Yan et al., 2019). Thus, there is high likelihood that some of the secondary metabolites extracted from medicinal plants are due to medicinal plants interaction with endophytes (David et al., 2015; Wu et al., 2021). Genomic based approach gives insight on various generic information, physiological, biological and evolutionary interactive processes of the plants (Sharma and Shrivastava, 2016). Understanding plants-endophytes physiological interactions and evolutionary relatedness can also assist in elucidating the relationship between certain metabolites and their therapeutic effectiveness (Atanasov et al., 2015). This can aid in the formulation of targets that focus solely on the relevant trends of secondary metabolites produced by plants and their associated endophytes (Li and Lou, 2018).

Comparing genomic traits of closely related endophytes with different functional roles obtained from the same plant is of most importance as this can help in determining their distinctive adaptability and evolutionary trend (Kaul et al., 2016). This can also help in comprehending different gene clusters that are responsible for different production of secondary metabolites (Kaul et al., 2016; Li and Lou, 2018). Metagenomics can as well aid in the discovery of novel biocatalysts and metabolic pathways by revealing previously unknown genomes (Bosamia et al., 2020). Genomic approach would help predicting whether metabolites produced by plants and microbes are due to novel gene clusters (Sinha et al., 2019; Atanas et al., 2021).

Different genomic techniques can be used to predict the direct biosynthetic gene clusters (BGCs) that encode the biosynthesis of secondary metabolites in microbial genomes (Keller, 2019). The archetypal cluster is the most significant as it contains the genes that are responsible for secondary metabolite synthesis (Rivera-Chávez et al., 2022). Following the identification of critical gene clusters for secondary metabolite production, it is imperative to discover limiting pathways for enhancing their biosynthesis, and thereafter eliminate them while modifying the pathways responsible for the metabolites of interest (Dutta et al., 2009). This could be through repetitive gene expression, deletion of unfavourable and competitive genes, and/or introduction of new genes required for better bioactive compound biosynthesis (Li and Lou, 2018; Pham et al., 2019). Deleting a competitive pathway helps in saving useful cellular resources for better and sufficient production of high-quality secondary metabolites (Pham et al., 2019; Li T. et al., 2020). This helps in creating conditions that are conducive for the gene of interest which is responsible for the production of the desired metabolite(s) (Venugopalan and Srivastava, 2015).

Synergism also can improve the possibilities of discovering novel compounds and boost therapeutic effectiveness of drugs. The possible identification of synergistic bioactive compounds under the umbrella of systems biology can usher this generation into the new era of drug discovery. It may be simple to combine and grow many endophytes and facilitate their interaction, which increases the likelihood of synthesising a variety of bioactive compounds (Sun et al., 2013). With medicinal plants, it may not be possible to combine two or more plants to produce a new bioactive compound. Nonetheless, the substances produced by the individual plants can be combined. Synergistic endophytes can then be used as biological elicitors for cultivation of medicinal plants in a controlled environment.

## 6 Endophytes as an important source of bioactive compounds

Dependence on medicinal plants for production of significant bioactive compounds for the development of novel drugs has a few drawbacks, including variation in secondary metabolites produced due to seasonal, spatial, and environmental conditions (Singh et al., 2018; Van Wyk and Prinsloo, 2020). Additionally, some medicinal plants have long generation time to reach maturity while some are strictly seasonal and thus unavailable during the off-season (Verma and Shukla, 2015; Isah, 2019). A considerable quantity of medicinal plants is required for the production of high yield secondary metabolites, however, due to rapid changes in climate and other factors, it is becoming more difficult to have a consistent supply of medicinal plants all year long. Finding a quick, economical, and reproducible solution for producing these compounds of pharmaceutical value is of utmost importance (Asmita and Sashirekha, 2016; Choi and Shin, 2020).

Novel strategies proposed for production of secondary metabolites should be more versatile, effective, and capable of promoting sustainable use of natural resources and as well support economic growth (Riisgaard et al., 2010; Dzoyem et al., 2013). Endophytes appear to be a feasible alternative for production of these key bioactive compounds. Endophytes are not only fast growing and inexhaustible, but they have also been classified as one of the sustainable natural resources since they encourage green processes (Choi and Shin, 2020). Thus, exploring them for different therapeutic properties is crucial. In addition, large-scale growth of endophytes in a fermenter under control conditions can result in an inexhaustible supply, which would ensure steady supply of the secondary metabolites. Furthermore, due to rapid development of mycelial biomass, production of bioactive compounds from fungal endophytes offers bigger benefits (Berde et al., 2020; Banyal et al., 2021).

Endophytes can produce secondary metabolites with various and distinct structures from that of their host plants, thus, suggesting a host-independent model of producing metabolites (Egamberdieva et al., 2017; Caruso et al., 2020). Endophytes have independent capacity to synthesize cyclic peptides of great pharmaceutical value (Ludwig-Müller, 2015). For instance, endophytes use dependent non-ribosomal peptide synthesis (NRPS) and independent NRPS pathways for additional production of bioactive compounds (Dhuldhaj and Pandya, 2021; Swayambhu et al., 2021). This has highlighted the potential of endophytes as a long-term pipeline for novel drug production and exploration to combat not only infectious diseases but also non-infectious illnesses like cancer, diabetes, arthritic conditions, and cardiac issues (Toghueo, 2019). Endophytes-based pharmaceuticals are attracting attentions due to their structural diversity, multitarget therapeutic actions and low cost of productions (Alvin et al., 2014). They are often found to be significantly safe, especially in cancer treatments, with minimal side effects on normal cells (Banyal et al., 2021). Substantial efforts have been carried out on the development of bioactive compounds from endophytic microbes and their application in the management of different medical conditions (**Tables 1**–**3**).

**Table 1:** Anticancer compounds produced by endophytic microorganisms.

Host Plants	Endophytes	Metabolites produced	References
*Capsicum annuum* L.	*Alternaria alternata*	Alkaloid, capsaicin	Devari et al. (2014)
*Nothapodytes foetida*	*Entrophospora infrequens*	Camptothecin	Puri et al. (2005)
*Panax ginseng*	*Burkholderia* sp.	Saponin: Ginsenoside Rg3	Singh and Dubey (2018)
*Taxodium distichum, Taxus wallichiana*	*Pestalotiopsis microspora*	Paclitaxel or Taxol	Berde et al. (2020)
*Panax ginseng*	*Burkholderia* sp.	Saponins: Ginsenoside Rg3, Ginsenoside Rg2	Fu et al. (2017)
*Zingiber officinale* (Ginger)	*Streptomyces aureofaciens*	4-arylcoumarins	Taechowisan et al. (2007)
*Cinnamomum cassia*	*Streptomyces cavourensis*		Vu et al. (2018)
*Camptotheca acuminata*	*Trichoderma atroviridae*	Camptothecin	Kumar et al. (2014)
*Taxus* sp.	*Fusarium chlamydosporum*	Kaempferol	Chaturvedi et al. (2014)
Mangrove	*Fusarium* sp. (No. DZ27	Beauvericin	Tao et al. (2015)
*Markhamia platycalyx*	*Aspergillus flocculus*	4hydroxymellein, 5hydroxymellein, diorcinol, botryoisocoumarin A	Tawfike et al. (2019)
*Anvillea garcinii*	*Fusarium chlamydosporium*	Fusarithioamide benzamide derivative	Ibrahim et al. (2016)
*Miquelia dentata* Bedd. (Icacinaceae)	*Lysinbacillus sp, Bacillus cereus*	Camptothecine	Shweta et al. (2013)
*Isodon eriocalyx*	*Streptomyces* sp. YIM66403	Anthracyclin	Li et al. (2015)
*Taxus cuspidata*	*Pericona* spp.	Periconicin A and B	Kim et al. (2004)
Mangrove	*Streptomyces* sp.	Indolocarbazoles	Xu et al. (2014)

**Table 2:** Antifungal compounds from endophytic microorganisms.

Plants	Endophytes	Metabolites produced	References
*Moringa oleifera*	*Nigrospora* sp. LLGLM003	Mullein, Griseofulvin, 8-dihydroramulosin	Zhao et al. (2012)
*Fragraea bodenii*	*Pestalotiopsis jesteri*	Jesterone	Li and Strobel (2001)
*Camellia sinensis*	*Pestalotiopsis fici*	Ficipyrone A	Liu et al. (2013)
*Taxus mairei*	*Aspergillus clavatonanicus*	Clavatol patulin	Zhang et al. (2008)
*Senna spectabilis*	*Phomopsis* sp.	Cytochalasin H	Chapla et al. (2014)
*Ephedra fasciculata*	*Xylaria* sp	Sordaricin (17)	Chen et al. (2018)
*Garcinia dulcis*	*Eutypella scoparia* PSU-D44	Scoparasin B	Pongcharoen et al. (2006)
*Glycyrrhiza uralensis*	*Bacillus atrophaeus*, *Bacillus mojavensis*	1,2 bezenedicarboxyl acid, Methyl ester, Decanodioic acid, bis(2-ehtylhexyl) ester	Mohamad et al. (2018)

**Table 3:** Antibacterial compounds from endophytic microorganisms.

Host plants	Endophytes	Metabolites produced	References
*Taxus cuspidata*	*Pericona* sp.	Periconicin A and B	Kim et al. (2004)
*Quercus* sp.	*Cytonaema* sp.	Cytonic acids A and B	Guo et al. (2000)
*Polygonum senegalense*	*Phomopsis longicolla*	Dicerandrol A, dicerandrol B, dicerandrol C	Lim et al. (2010)
*Urospermum picroides*	*Ampelomyces* sp.	Altersolanol A	Aly et al. (2008)
*Salicornia bigelovii Torr*	*Fusarium tricinctum*	Enniatin B	Zhang et al. (2015)
*Ephedra fasciculata*	*Fusarium tricinctum*	Fusartricin	Zhang et al. (2015)
*Kennedia nigriscans*	*Streptomyces* NRRL 30562	Munumbicins	Castillo et al. (2002)

### 6.1 Fungal and bacterial endophytes

Endophytic fungi are essential sources of valuable bioactive compounds linked to most of the commonly used antibiotic and anticancer drugs, among which are vinblastine, camptothecin, hypericin, paclitaxel, diosgenin and podophyllotoxin (Kumar et al., 2014; Gouda et al., 2016). The penicillenols, derived from *Penicillium* sp., are cytotoxic to a variety of cell lines. Taxol, derived from the endophytic fungi *Taxomyces andreanae*, is the most potent and successful anticancer medication to date. Clavatol (*Torreya mairei*), sordaricin (*Fusarium* sp.), jesterone (*Pestalotiopsis jesteri*), and javanicin (*Chloridium* sp.) are all known to have potent antibacterial and antifungal activities against a wide range of pathogens. Pestacin, derived from *Microsporum* sp. has very powerful antioxidant properties (Kumar et al., 2014; Gouda et al., 2016). *Pestalotiopsis neglecta* BAB-5510 and *Cupressus torulosa* are endophytic fungi considered to be a promising source of phenols, flavonoids, terpenoids, alkaloids, and tannins (Pimentel-Elardo et al., 2010). Capsaicin, a bioactive compound prevalent in red and chilli peppers, is a pain reliever and an anti-cancerous agent applied in the treatment of human malignancy. Capsaicin is produced by *Alternaria alternata*, an endophytic fungus identified from *Capsicum annum* (Zhao et al., 2011a).

Moreover, actinomycetes particularly *Streptomyces*sp. sp., have been identified as a promising source of bioactive metabolites (Zhao et al., 2011b; Golinska et al., 2015). To date, over 140 genera of actinomycetes have been identified, but only a small fraction of them produce the majority of known essential antibiotics (Pimentel-Elardo et al., 2010). *Streptomyces* are known to be rich in many bioactive compounds such as munumbicins (A and B), naphthomycin (A and K), clethramycin, coronamycin, cedarmycin (A and B), saadamycin, and kakadumycins. *Streptomyces rochei* CH1, an endophytic actinomycete of *Cinnamomum* sp., showed significant antibacterial activity against various pathogens like *Aeromonas caviae*, *Vibrio parahemolyticus* and *Pseudomonas aeruginosa* (Roy and Banerjee, 2015).

## 7 Metabolite yield enhancement strategies by the endophyte

In general, microorganisms are susceptible to genetic manipulation for desired performance. Different techniques have been successfully applied in the attempt to enhance secondary metabolite production by endophytes, and these include strain improvement, mutagenesis, co-cultivation, and medium optimization (Kumar et al., 2014) (**Table 4**).

**Table 4:** Examples of successful strain improvement strategies.

Compound	Type of strain improvement strategy	Original organisms	Yield (folds)	References
6′-deoxy-bleomycin Z	UV, mutagenesis and ribosome engineering	*Streptomyces flavoviridis* G-4F12	7.00	Zhu et al. (2018)
Vinblastine	gamma irradiation	*Alternaria alternata*	3·98	El-Sayed (2021)
Glycoside digoxin	Gamma irradiation	*Epicoccum nigrum*	5.00	El-Sayed et al. (2020)
Taxol	UV irradiation, Nitrous acid Ethidium bromide	*Aspergillus* sp.	1.10	Khan et al. (2020)
Paclitaxel	UV, gamma irradiation	*Aspergillus fumigatus*, *Alternaria tenuissima*	1.22 1.24	El-Sayed et al. (2019)
Tanshinone IIA	UV, NaNO_2_	*Emericella foeniculicola* TR21	1.46	Ma et al. (2011)
Tanshinone IIA	Genomic shuffling	*Emericella foeniculicola*	11.07	Zhang et al. (2018)
Lovastatin	Genomic shuffling	*Fusarium* sp. ALAA20	11.55	El-Bondkly et al. (2021)

### 7.1 Strain improvement

The process of strain improvement involves genetically modifying microbial strains with the aim of enhancing their capabilities for a wide range of applications. This involves interactive genetic modifications and fermentation techniques (Barrios-Gonzalez et al., 2003; Ademakinwa et al., 2017). The process would not only bring about elevated product yield, but also focus on enhancing and creating new genetic traits, such as eliminating unwanted co-metabolites, improving nutrient utilization, and modifying cellular morphology to enhance oxygen transfer during fermentation or to facilitate downstream processing (Barrios-Gonzalez et al., 2003). It also offers several advantages such as modifying product ratios, by-products removal, product excretion enhancement, increasing tolerance to high product concentrations, reduction in fermentation time, and high production of native or foreign products through genetic recombination (Li P. et al., 2012).

### 7.2 Mutagenesis

The classical technique of induced mutagenesis has been successfully applied in strains improvement and increasing production of microbial metabolites that are of commercial significance. This approach has contributed significantly to the progress made in strain improvement (El-Sayed et al., 2019). Mutagens are chemical compounds such as hydroxylamine, nitrosoguanidine, methyl methanesulfonate, ethyl methanesulfonate, nitrosoguanidine, ethyl methyl sulfonate, sodium nitrite and diethyl sulfate, as well as physical forms of radiation such as ultraviolet (UV) light or X-rays. These mutagens have the ability to cause permanent and inheritable changes (mutations) in the microbial genome (Ma et al., 2011; Khan et al., 2020). When selecting for improved mutants, there are two approaches: random selection for the desired feature or target selection based on specific characteristics that differ from the previous genotype of interest (Zhang et al., 2018).

### 7.3 Genetic engineering

In order to enhance secondary metabolites, various genetic methods are employed in strain improvement. These include amplification of secondary metabolite gene clusters, cloning of regulatory genes, inactivation of competing pathways, disruption or amplification of regulatory genes, manipulation of secretory mechanisms, and expression of a suitable heterologous proteins. These are accomplished through application of different techniques namely; (i) transposition mutagenesis, (ii) target deletions and duplications by genetic engineering and (iii) genetic recombination by protoplast fusion (Genilloud, 2018). Recent additions to these techniques include genomics, transcriptome, proteomics, metabolic engineering analyses, and whole genome shuffling (Adrio and Demain, 2006).

### 7.4 Molecular breeding techniques

Techniques of DNA shuffling and molecular breeding involve *in vitro* homologous recombination i.e., mimicking the natural process of recombination (Gunatilaka, 2006). These methods include both the recombination of DNA fragments and the controlled introduction of point mutations at a low rate (Bode et al., 2002; Paranagama et al., 2007). Genome shuffling of similar genes from different species or strains yields significant improvements, unlike site-directed mutagenesis which is more expensive and time consuming (Patten et al., 1997). An updated approach of this method involves breeding a population with high genetic variability i.e., DNA family shuffling. By starting with four cephalosporinase genes, this method led to a significant increase in cephalosporinase activity ranging from 240 to 540 folds (Raimi and Adeleke, 2021). In contrast, when each of these genes was shuffled independently, only an eight-fold improvement was achieved. Whole genome shuffling is a new approach for strain improvement that combines the benefit of multi-parental crossover enabled by DNA shuffling with the recombination of whole genomes. According to Halpin et al. (2001), this technique increased the production of tylosin in *Streptomyces fradiae*.

### 7.5 Co-culturing

Microbial co-cultivation, also called mixed fermentation, refers to the process of cultivating two or more microorganisms in a single confined environment. When endophytes are isolated and cultured in axenic monocultures to produce the desired product, repeated culturing may lead to the loss of access to the specific microenvironment of the host plant to which the endophyte has adapted. This loss of access may result in the silencing of the endophyte’s secondary metabolite genes, and standard culture conditions may not be sufficient to activate the expression of these genes (Somjaipeng et al., 2016; Venugopalan et al., 2016). Moreover, typical cultural methods might not be sufficient to activate the hidden biosynthetic gene clusters observed in endophytes, which could lead to either redundancy in terms of identifying new metabolites or a decrease in the amount of generated metabolites. Based on whole genome sequencing techniques, the quantity of genes encoding biosynthetic enzymes in various fungi and bacteria surpasses the number of secondary metabolites that are currently recognized in these microorganisms.

Co-culturing is regarded as a highly effective ecological method that involves replicating the natural environment of the host in a laboratory setting, which includes both competition and coexistence with other endophytic communities. As shown in **Table 5**, this approach can have a positive effect on the production of particular metabolites. By fermenting a mixture of microbes, it is possible to stimulate the expression of silent biosynthetic genes that may result in the creation of novel secondary metabolites, often through the use of signaling molecules such as auto-regulators, quorum-sensing molecules and siderophores (Bertrand et al., 2014; Marmann et al., 2014). A different interpretation proposes that this phenomenon may be connected to the creation of enzymes that stimulate the production of the metabolite precursor generated by the producing strain leading to the active metabolites (Abdelmohsen et al., 2015). Alternatively, the inducing strain might prompt epigenetic changes in the producing strain. To fully comprehend these interactions between endophytes, as well as between endophytes and host plants, a thorough knowledge of their ecological relationships is necessary (Wakefield et al., 2017).

**Table 5:** Effect of co-cultivation strategy on product yield and discovery of novel metabolites from endophytes.

Products	Original species	Co-culturing strain	Yield enhancement	References
Luteoride D pseurotin G 11-*O*-methylpseurotin	*Aspergillus fumigatus* MR2012	*Streptomyces leeuwenhoekii* C34 & C58	New metabolites	Wakefield et al. (2017)
Taxol	*Paraconiothyrium* SSM001	*Alternaria alternate*	3 fold	Soliman and Raizada (2013)
Taxol	*Fusarium*	*Taxus* suspension cells	38 fold	Li et al. (2009)
Enniatins A1 and B1	*Fusarium tricinctum*	*Bacillus subtilis* 168 trpC2	78 fold	Wätjen et al. (2009)
Enniatins A1, B1, and B (cyclic depsipeptides)	*Bacillus subtilis* 168 trpC2	*Fusarium tricinctum*	78 fold	Ola et al. (2013)
Taxol	*Taxus chinensis* var. *Taxus cuspidate*	*Fusarium mairei*	38 fold	Li et al. (2009)
Cercosporin	*Cercospora sp*	*Bacillus velezensis* B04 *Lysinibacillus* sp. B15	4.89 fold	Zhou et al. (2021)
Hypocrellin A	*Shiraia* sp	*P. fulva*, *P. putida*, and *P. parafulva*	3.25 fold	Ma et al. (2019)
Camptothecine	*Colletotrichum fructicola* UK1	*Corynespora cassiicola* SUK2	4 fold	Bhalkar et al. (2016)
Bisvertinol, Koninginin A, Mevastatin, 1-naphthylacetic acid	*Trichoderma atroviride* SG3403	*Bacillus subtilis* 22	Increased 2^0^ metabolites	Tang et al. (2020)
Citrinin	*Aspergillus sydowii* EN-534	*Penicillium citrinum* EN-535	Novel ten citrinin analogues	Wang et al. (2022)
Chrysophaein, resveratrol, chrysophanol, emodin, physcion	*Fusarium* sp.	*Aspergillus* sp.	3.85-fold	Ding et al. (2018)

### 7.6 Medium optimization

The initial phase in carrying out a substantial metabolite synthesis is medium optimization, which remains a highly scrutinized process and has several challenges associated with it. In the past, traditional methods were used for media optimization which were costly, time-consuming, and involved numerous experimental procedures with less accuracy. However, with the advent of sophisticated mathematical and statistical techniques, such as artificial neural networks and genetic algorithms, media optimization has become more dynamic, productive, cost-effective, and dependable in yielding results (Franco-Lara et al., 2006). The production of Zofimarin, a potent antimicrobial agent derived from *Xylaria* sp. Acra L38, was increased by 8 times through the optimization of carbon and nitrogen sources by utilizing orthogonal array and Plackett Burman designs (Chaichanan et al., 2014). Similarly, in the case of taxol, a renowned anticancer bioactive substance obtained from *Fusarium mairei* UH23, the yield was increased by 10.2% through optimization of carbon and nitrogen sources and the fermentation period, making use of single factor experiment (Wen-yi, 2008).

Camptothecin, which is a prominent anticancer agent of the 21st century, is produced by *Trichoderma atroviride* (Egbuna et al., 2020). Through the use of the single factor method, by optimizing the medium composition, fermentation time (including elicitor and adsorbent addition), the production of camptothecin was increased by 50 to 75 times (Pu et al., 2013). Optimisation of medium parameters has been proven to enhance production of secondary metabolites by microorganisms (**Table 6**). For instance, modifying the carbon and nitrogen sources, and trace elements through the use of factorial design and RSM, the yield of exopolysaccharide (EPS) from *Berkleasmium* sp. Dzf12 was also improved by 6.29 times (Li J. et al., 2012).

**Table 6:** Effect of changed parameters on yield of metabolites of different microorganisms.

Product	Producing Strain	Optimized parameters	Yield (folds)	Reference
Vitexin	*Dichotomopilus funicola* Y3	Medium composition	4·59	Gu et al. (2018)
Epothilone B	*Aspergillus fumigatus*	Sucrose, tryptone and incubation time	2.8 - 3.0	El-Sayed (2021)
Palmarumycin C_13_	*Berkleasmium* sp. Dzf12	Glucose, peptone and yeast extract conc opt	2.50	Zhao et al. (2013)
Exo-polysaccharide	*Berkleasmium* sp. Dzf12	C and N sources	6.29	Li J. et al., (2012)
Beauvericin	*Fusarium redolens* Dzf2	C andN sources, initial pH	1.27	Tao et al. (2015)
Zofimarin	*Xylaria* sp. Acra L38	C and N sources, temperature, fermentation period	8.00	Chaichanan et al. (2014)
Antibiotics	*Streptosporangium*	Aeration (gas flow), agitation system and stirrer speed	20.00	Pfefferle et al. (2000)
Taxol	*Nodulisporium sylviforme*	pH, temperature, agitation rate,	1.15	Zhao et al. (2011b)
Lovastatin	*Aspergillus terreus*	B-group vitamins, MgSO_4_ and sodium acetate.	42.00	Ravuri and Shivakumar, (2020a)
Lovastatin	*Meyerozyma guilliermondii*	Zinc sulphate, histidi, tween-8 and B-group vitamins	11.40	Ravuri and Shivakumar, (2020b)
Palmarumycin C13	*Berkleasmium* sp. Dzf12	Metal ions	6.00	Mou et al. (2013)
Camptothecin	*Fusarium oxysporum* NFX06	Glucose, peptone and MgSO_4_	1.02	Musavi et al. (2015)
Camptothecin	*Fusarium solani*	Initial pH, temperature and agitation speed	152.00	Venugopalan et al. (2016)
Camptothecin	*Trichoderma atroviride* LY357	Medium composition, fermentation time, pH, temperature, agitation rate	50 - 75	Pu et al. (2013)
Vincamine	XM-J2	Medium composition and pH	1.40	Yin and Sun (2011)

### 7.7 Limitations of secondary metabolites production by the endophytes

It is a well-established concept that microbial metabolisms are tightly connected to the selection of culture media and other conditional parameters (Kusari et al., 2012). Considering the scale of complexity in the endophyte-endophyte and endophyte-plants interactions in their ecological niche, choice of appropriate growth support media and varying morphological and physiological responses of the endophytic microbes in different culture media remain a major pitfall. For instance, Paranagama et al. (2007) demonstrated a significant switch in the secondary metabolites produced by endophytic *Paraphaeosphaeria quadriseptata* after the tap water used in the preparation of the growth media was substituted with distilled water. The production of radicicol instead of chaetochromin A was also reported when the agar medium was replaced with broth during *in vitro* laboratory culture of the *Chaetomium chiversii*. Inefficiency of endophytes in the biosynthesis of certain molecules of interest when grown in different culture media in laboratories creates a wide gap in the understanding of full potential of the endophyte biosynthetic capability (Toghueo et al., 2020).

In addition, when endophytes are isolated from respective host plants to be screened for their biosynthetic active metabolite capabilities, there might be substantial loss of great potentials which were built on inherent ecological chemical interactions while functioning within their niche. Most isolated endophytes are often discarded as incompetent endophytes after *in vitro* screening based on the observed potential as individual microbe. This results in losing out on whole lots of desired important metabolites which can only be synthesized optimally in synergy with endophytic community while interacting with the host plant tissues (Kusari et al., 2012). Moreover, due to undiscovered genes or unknown enzyme pathways, endophytes heterologous gene expression makes it even more difficult to precisely link secondary metabolite biosynthetic pathways with specific enzymatic activities (Medema et al., 2021). Understanding the full potential of endophytes in biosynthesis of secondary metabolites could be further hindered by linear gene functions, gene co-expression, and gene duplication as well as various kinetics and characteristics of enzymes involved (Isah et al., 2018). As much as omics techniques like transcriptomics, metabolomics, and proteomic analyses are helpful in revealing the secondary metabolite-producing and/or non-metabolites producing microbes, they are not without limitations (Paz et al., 2017). It is still a difficult task to deeply comprehend the relationship between proteomics and metabolomics patterns in an endophyte (Isah et al., 2018).

### 7.8 Prospects of the endophytes-medicinal plants secondary metabolite production

The major limiting factors in the supply of medicinal plants for drug discovery include conservation regulatory measures, off-season periods and environmental variations, pathogens attack, heavy metal contamination, and low production of secondary metabolites. Hence, a deep understanding of how endophytes enhance and regulate secondary metabolite production by the host plants is vital to the desired up-scaling of therapeutic values of medicinal plants, because endophytes are the primary drivers of the host plant resistance to abiotic and biotic stress. The interaction between the two symbiotic partners is informed by very strong synergy rooted from their genetic makeup (Mengistu, 2020).

Understanding the balance between plant defense and microbial virulence may also offer better outcomes in unlocking the full potential of medicinal plants and endophytes for secondary metabolite production. Investigating possibility of chemical elicitors influence on the full capacity of endophytes in biosynthesis of secondary metabolite appears to be a viable solution. Chemical elicitors, for instance, could induce epigenetic changes in endophytes, and thereby activate most silent genes (Pacheco-Tapia et al., 2022). Xue et al. (2023) have also mentioned that the use of small molecular weight compounds such as proteasome and histone deacetylase could be useful in rectifying limitations regarding inadequate production of secondary metabolites by endophytes.

Eliciting increased bioactive secondary metabolite production in medicinal plants by the endophytes has been demonstrated to be an effective approach. Combination of endophytic genes that encode a specific biosynthetic pathway for a secondary metabolite via hybrid pathways could also be effective in overcoming gene-based secondary metabolite constraints. For example, the synthesis of terpenoid secondary metabolites, which have anti-cancer properties, from actinomycetes bacteria was successfully increased with a biosynthetic hybrid isoprenoids (Gallagher et al., 2010). Lastly, chemo-taxonomic information and molecular phylogenetic data can also be used to better understand the relationship between certain metabolites and their therapeutic effectiveness (Atanasov et al., 2015). This would aid the formulation of particular targets that focus solely on the relevant trends of secondary metabolites produced by plants and their associated microbes (Li and Lou, 2018).

## Author contributions

ST, KA, MR, SA, and RA contributed to the design of the study. The first draft of the manuscript was written by ST. ST, SH and KA wrote different sections of the manuscript. All authors contributed to the article and approved the submitted version.
